# Robust Fuzzy Logic Stabilization with Disturbance Elimination

**DOI:** 10.1155/2014/171597

**Published:** 2014-08-06

**Authors:** Kumeresan A. Danapalasingam

**Affiliations:** ^1^Department of Control & Mechatronics Engineering, Faculty of Electrical Engineering, Universiti Teknologi Malaysia, UTM Skudai, 81310 Johor, Malaysia; ^2^UTM Centre for Industrial and Applied Mathematics, Universiti Teknologi Malaysia, UTM Skudai, 81310 Johor, Malaysia

## Abstract

A robust fuzzy logic controller is proposed for stabilization and disturbance rejection in nonlinear control systems of a particular type. The dynamic feedback controller is designed as a combination of a control law that compensates for nonlinear terms in a control system and a dynamic fuzzy logic controller that addresses unknown model uncertainties and an unmeasured disturbance. Since it is challenging to derive a highly accurate mathematical model, the proposed controller requires only nominal functions of a control system. In this paper, a mathematical derivation is carried out to prove that the controller is able to achieve asymptotic stability by processing state measurements. Robustness here refers to the ability of the controller to asymptotically steer the state vector towards the origin in the presence of model uncertainties and a disturbance input. Simulation results of the robust fuzzy logic controller application in a magnetic levitation system demonstrate the feasibility of the control design.

## 1. Introduction

One of the key challenges in solving practical controller design problems is the availability of an accurate mathematical model of a plant. Due to the presence of unmodelled nonlinearities, parameter uncertainties, measurement errors, actuator errors, and external disturbances in a system to be controlled, the derivation of a reliable mathematical model for control purposes is a demanding task. In cases where an acceptable mathematical model of a plant does not exist or is difficult to be obtained, the fuzzy logic controller is often useful [1, 2]. A unique feature of fuzzy logic controllers is that it enables a natural framework to incorporate linguistic descriptions of a system and control rules from human experts [3, 4]. A fuzzy logic controller is often advantageous in cases where field engineers or operators are able to furnish linguistic fuzzy control rules or linguistic fuzzy descriptions about a system to be controlled. Compounded by the fact that it is a nonlinear controller based on the model-free design, fuzzy logic controllers have been favourable in numerous household and industrial applications.

Some previous works on fuzzy logic control are reviewed here. The problem of adaptive fuzzy decentralized fault-tolerant control is investigated for a class of nonlinear large-scale systems in [5]. The proposed control design applies a fuzzy logic system to approximate unknown nonlinear functions that exist in the plant and a fuzzy adaptive observer to estimate unmeasured states. By choosing appropriate design parameters, it is shown that all closed-loop signals are bounded and that tracking errors converge to a small neighborhood of zero. In [6], an adaptive fuzzy robust output feedback control problem is considered for nonlinear systems that possess unstructured uncertainties, unknown dead zone, and dynamics uncertainties. By combining a backstepping technique with a nonlinear small-gain approach, the proposed controller demonstrates semiglobal uniform ultimate boundedness for all closed-loop solutions. An *H*_*∞*_ output-feedback fuzzy controller is designed for a class of discrete-time fuzzy systems with randomly occurring infinite distributed delays and channel fadings in [7]. By utilizing the cone complementarity linearization algorithm, the closed-loop Takagi-Sugeno fuzzy control system is proven to be exponentially mean-square stable, and the disturbance rejection attenuation is constrained to a given level by means of the *H*_*∞*_ performance index. In [8], the stabilization of a class of discrete-time Takagi-Sugeno (T-S) fuzzy systems with stochastic perturbation and time-varying state delay is studied. In the research, a fuzzy Lyapunov-Krasovskii function is constructed, and some examples are provided to illustrate the effectiveness of the proposed methods. An adaptive fuzzy control strategy with guaranteed convergence of an optimal fuzzy approximation error is presented for a class of uncertain nonlinear systems in the general Brunovsky form in [9]. The authors prove that the closed-loop system achieves partially asymptotic stability under a certain selection of control parameters and report a high-precision tracking performance through simulation studies.

Conventional controllers are required to be tuned by adjusting its controller gains to obtain acceptable control performances. Similarly, a fuzzy logic controller needs to be tuned until a satisfactory control performance is achieved. A fuzzy logic controller can be tuned by modifying its fuzzy control rules, membership functions, and scaling gains. In this paper, elements of some of the membership functions are chosen as the tuning parameter. While a similar approach is undertaken in [10, 11], asymptotic stability is not achieved therein. In this work, the tuning parameter of the proposed fuzzy logic controller is adapted for asymptotic stability. The contribution of this research is the development of an asymptotically stable fuzzy logic controller that is robust against model uncertainties and a disturbance input whose measurements are not available.

A fuzzy logic control problem is addressed for a class of nonlinear dynamical control systems in Section 2. In the same section, the components of a fuzzy logic controller are described. This is followed by Section 3 that covers the design of a robust fuzzy logic controller. To confirm the performance of the proposed controller, simulation results are presented and analyzed in Section 4.  Section 5 concludes the paper.

## 2. Preliminaries

This work concerns the problem of robust fuzzy logic stabilization for general nonlinear control systems of the form

(1) x˙1=x2⋮x˙n−1=xnx˙n=f(x)+g(x)u+d(t), x∈Rn,  u∈U,  n=2,3,

where **x** is the state vector, *f* : *R* ^*n*^ → *R* and *g* : *R* ^*n*^ → *R*, *g*(**x**) ≠ 0 are continuous functions, *u* is the control input taking values in a compact set *U* ⊂ *R*, and  *d*(*t*) is a continuous function denoting unmodelled dynamics and disturbances. The control system is subject to model uncertainties; that is,

(2) $$\begin{array}{c} f\left(x\right)=f_{0}\left(x\right)+f_{\Delta }\left(x\right),\\ g\left(x\right)=g_{0}\left(x\right)+g_{\Delta }\left(x\right), \end{array}$$

where *f*_0_(**x**) and *f*_Δ_(**x**) (*g*_0_(**x**) ≠ 0 and *g*_Δ_(**x**)) are known nominal and unknown uncertain functions, respectively. The robust fuzzy logic stabilization problem involves the design of a dynamic feedback controller *k* : *R* ^*n*^ × *R* → *U* such that the origin in *R* ^*n*^ is robustly stable with respect to the trajectories of the closed-loop system

(3) x˙1=x2⋮x˙n−1=xnx˙n=f(x)+g(x)k(x,t)+d(t).

Here, the term robustness refers to the insensitivity of a controller's performance with respect to modelling errors *f*_Δ_(**x**) and *g*_Δ_(**x**) and a persistent disturbance *d*(*t*).

Fuzzy logic controllers have been successfully applied in many commercial products and industrial systems (see, for instance, [12–15]) and one contributing factor for its effectiveness is that it is inherently a nonlinear controller [16–19]. Given its good reputation in control engineering, it is desired to include a fuzzy logic controller in the design of a robustly stabilizing *k*(**x**, *t*).

### 2.1. Fuzzy Logic Controller

**Figure 1:**
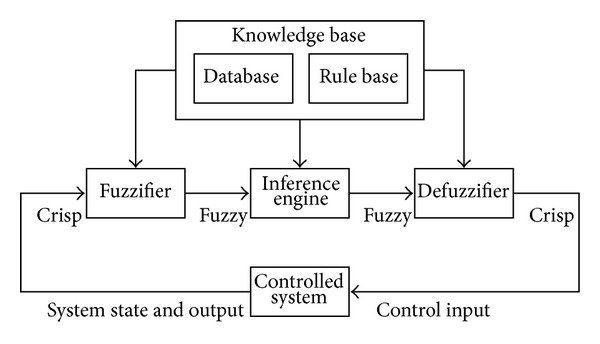
Fuzzy logic controller configuration.

A typical fuzzy logic controller consists of fuzzifier, knowledge base, inference engine, and defuzzifier as shown in Figure 1. The fuzzy logic controller is a feedback

(4) $$\begin{array}{c} k_{fl}:R^{n}⟶U. \end{array}$$

The fuzzifier transforms a real or crisp **x** = [*x*_1_ ⋯ *x*_*n*_]^*⊤*^ into a fuzzy set through the fuzzification operation. A fuzzy set *F* is a set of ordered pairs of  **x** and its value of membership function *μ*_*F*_(**x**); that is, *F* = {(**x**, *μ*_*F*_(**x**))} ⊂ *R* ^*n*^ × [0,1] [20]. In fuzzy control applications, it is common to convert a crisp value **x**_0_ of **x** to a fuzzy singleton, that is, a fuzzy set where *μ*_*F*_(**x**_0_) = 1 and *μ*_*F*_(**x**) = 0 for **x** ≠ **x**_0_ [10, 11, 17].

The knowledge base comprises a database that provides necessary definitions to ensure a proper functioning of the controller and a rule base that contains a set of fuzzy control rules [21]. A fuzzy control rule provides a convenient way to represent control goals and policies of field experts and has the following form:

(5)  Rj:If  x1  is  A1j  and  …  and  xn  is  Anj  then  ufl  is  Bj, j=1,2,…,M,

where *x*_*i*_ and *u*_*fl*_ are linguistic variables representing *i*th input of the controller and fuzzy logic control input, respectively, *A*_*i*_^*j*^ and *B* ^*j*^ are linguistic values of the linguistic variables *x*_*i*_ and *u*_*fl*_, respectively, and *M* is the number of fuzzy control rules.

Each fuzzy control rule (5) is expressed as a fuzzy implication *R* ^*j*^ in the inference engine. A fuzzy implication is a fuzzy relation defined as

(6) μRj(x,ufl):=μA1j  and  …  and    Anj→Bj(x,ufl)=μA1j×⋯×Anj→Bj(x,ufl)=μA1j×⋯×Anj(x)⟶μBj(ufl).

In (6), the logical operator “and” is implemented as a fuzzy conjunction and is given by

(7) $$\begin{array}{c} \mu_{A_{1}^{j}\times \cdots \times A_{n}^{j}}\left(x\right)=\mu_{A_{1}^{j}}\left(x_{1}\right)*\cdots *\mu_{A_{n}^{j}}\left(x_{n}\right), \end{array}$$

where the symbol ∗ represents the triangular norm. Some commonly used triangular norms are intersection, bounded product, drastic product, and algebraic product that is defined as

(8) $$\begin{array}{c} \mu_{A_{1}^{j}}\left(x_{1}\right)*\cdots *\mu_{A_{n}^{j}}\left(x_{n}\right)=\mu_{A_{1}^{j}}\left(x_{1}\right)\mu_{A_{2}^{j}}\left(x_{2}\right)\cdots \mu_{A_{n}^{j}}\left(x_{n}\right). \end{array}$$

Many types of fuzzy implication can be found in the literature such as min⁡ operation rule, product operation rule, bounded product operation rule, drastic product operation rule, arithmetic rule, maximin rule, standard sequence, Boolean implication, Gödelian logic, and Goguen implication [2, 10, 20]. In this paper, the product operation rule of fuzzy implication is of particular interest, where, from (6), (7), and (8),

(9) μRj(x,ufl)=μA1j×⋯×Anj(x)⟶μBj(ufl)=μA1j×⋯×Anj(x)μBj(ufl)=μA1j(x1)μA2j(x2)⋯μAnj(xn)μBj(ufl).

By applying the sup-star compositional rule of inference on a fuzzy singleton *F* (output of the fuzzifier) and a fuzzy implication *R* ^*j*^, a fuzzy set *F*∘*R* ^*j*^ ⊂ *R* × [0,1] is obtained for each fuzzy control rule (5) as follows:

(10) $$\begin{array}{c} \mu_{F\circ R^{j}}\left(u_{fl}\right)=\underset{x\in R^{n}}{\sup}\left[\mu_{F}\left(x\right)*\mu_{R^{j}}\left(x,u_{fl}\right)\right], \end{array}$$

where ∘ is a compositional operator and “star” or ∗ denotes the triangular norm. Note that, by using algebraic product and (9), (10) becomes

(11) μF∘Rj(ufl) =sup⁡x∈Rn[μF(x)μA1j(x1)μA2j(x2)⋯μAnj(xn)μBj(ufl)].

The defuzzifier maps a fuzzy control action obtained in the inference engine to a crisp fuzzy logic control input *u*_*fl*_ ∈ *U*. Some defuzzification strategies include maximum, center-average, modified center-average, mean of maximum, and center of area defuzzifiers [11, 22]. In particular, the center-average defuzzifier is expressed as

(12) $$\begin{array}{c} u_{fl}=\frac{\sum_{j=1}^{M}u_{0}^{j}\mu_{F\circ R^{j}}\left(u_{0}^{j}\right)}{\sum_{j=1}^{M}\mu_{F\circ R^{j}}\left(u_{0}^{j}\right)}, \end{array}$$

where *u*_0_^*j*^ = arg max⁡_*u*_*fl*_∈*U*_[*μ*_*B*^*j*^_(*u*_*fl*_)]. By assuming that max⁡_*u*_*fl*_∈*U*_[*μ*_*B*^*j*^_(*u*_*fl*_)] = 1 and implementing (11), (12) can be rewritten as [12]

(13) $$\begin{array}{c} k_{fl}\left(x\right)=\frac{\sum_{j=1}^{M}u_{0}^{j}\left(\prod_{i=1}^{n}\mu_{A_{i}^{j}}\left(x_{i}\right)\right)}{\sum_{j=1}^{M}\left(\prod_{i=1}^{n}\mu_{A_{i}^{j}}\left(x_{i}\right)\right)}. \end{array}$$

To effectively control a plant, controllers are needed to be tuned either manually or automatically until a satisfactory performance is obtained [23–25]. While the tuning parameters of most of the controllers are gains, a fuzzy logic controller can be tuned by adjusting its fuzzy control rules, membership functions, and scaling gains [26]. In this paper, the tuning parameter of the fuzzy logic controller *k*_*fl*_(**x**) is *ξ* ∈ *R* ^*M*^ in the following representation of (13):

(14) $$\begin{array}{c} k_{fl}\left(x\right)=\psi \left(x\right)\xi , \end{array}$$

where

(15) ψ(x)=1∑j=1M(∏i=1nμAij(xi)) ×[(∏i=1nμAi1(xi))⋯(∏i=1nμAiM(xi))],ξ=[u01u02⋯u0M]⊤.

## 3. Fuzzy Logic Controller Design

In this section, a dynamic feedback controller is designed in two stages to achieve the control objective described in Section 2. Firstly, a preliminary feedback law that compensates the nonlinear terms *f*(**x**) and *g*(**x**) in (1) is proposed based on the known nominal functions *f*_0_(**x**) and *g*_0_(**x**). Secondly, a dynamic fuzzy logic controller is designed by means of a linear time-varying system to compensate for the unknown model uncertainties *f*_Δ_(**x**) and *g*_Δ_(**x**) and disturbance *d*(*t*).

Consider the following expression from (1):

(16) $$\begin{array}{c} {\dot{x}}_{n}=f\left(x\right)+g\left(x\right)u+d\left(t\right). \end{array}$$

To compensate for the nonlinear terms *f* and *g* in (16), a preliminary control law is chosen as follows:

(17) $$\begin{array}{c} u=\frac{v-f_{0}\left(x\right)}{g_{0}\left(x\right)}, \end{array}$$

where *v*  is an additional control input to be designed. It is easy to show that the control input *v* needed to achieve ${\dot{x}}_{n}=0$ is

(18) $$\begin{array}{c} v^{ff}=f_{0}\left(x\right)-\frac{g_{0}\left(x\right)}{g\left(x\right)}\left(f\left(x\right)+d\left(t\right)\right). \end{array}$$

Feedforward control law *v* ^*ff*^ (18) applied in (17) is capable of keeping **x**(*t*) identically at zero if the initial conditions are set as *x*_1_(0) = ⋯ = *x*_*n*_(0) = 0. Since *v* ^*ff*^ depends on the uncertain *f*, *g* and unknown *d*(*t*), control law (18) is not directly implementable.

To asymptotically regenerate *v* ^*ff*^, it is first assumed that (18) can be viewed as an output generated by the linear time-varying system

(19) $$\begin{array}{c} {\dot{\xi }}^{ff}=\left(F+G\psi \left(x\right)\right)\xi^{ff},\\ v^{ff}=f_{0}\left(x\right)+\psi \left(x\right)\xi^{ff}, \end{array}$$

where *F* is an *M* × *M* Hurwitz matrix, *G* is an *M* × 1 vector such that the pair (*F*, *G*) is controllable, *ψ*(**x**) is from (14), and *ξ* ^*ff*^ ∈ *R* ^*M*^ is the state variable. Based on assumption (19), a dynamic fuzzy logic controller utilizing fuzzy logic controller *k*_*fl*_ (14) is proposed resulting in

(20) $$\begin{array}{c} \dot{\xi }=\left(F+G\psi \left(x\right)\right)\xi +Gw,\\ v=f_{0}\left(x\right)+\psi \left(x\right)\xi +w, \end{array}$$

where *ξ* ∈ *R* ^*M*^ is the controller state and *w* is a function to be determined. Note that the fuzzy logic controller is implemented in (20) to estimate the uncertain and unknown terms in (18). In order to formally prove that control laws (17) and (20), that is,

(21) $$\begin{array}{c} \dot{\xi }=\left(F+G\psi \left(x\right)\right)\xi +Gw,\\ v=f_{0}\left(x\right)+k_{fl}\left(x\right)+w,\\ k\left(x,t\right)=\frac{v-f_{0}\left(x\right)}{g_{0}\left(x\right)}, \end{array}$$

asymptotically stabilize control system (3), the following theorem is presented.

**Theorem 1 .**

There exists a function *w* such that control system (3) with dynamic feedback controller (21) is asymptotically stable.

**Proof**

Even though only steps of proof for cases *n* = 2 and *n* = 3 are shown, a similar approach can be taken for a system of any order.

(*1) Case n* = 2. A new vector of state variables is defined as

(22) $$\begin{array}{c} \eta_{2}=\left[\begin{array}{c} e_{\xi }+\frac{g_{0}}{g}x_{2}G\\ x_{1}\\ x_{2}+k_{1}x_{1} \end{array}\right], \end{array}$$

where

(23) $$\begin{array}{c} e_{\xi }=\xi -\xi^{ff} \end{array}$$

and *k*_1_, *k*_2_ are tuning parameters of the feedback law

(24) $$\begin{array}{c} w=-k_{2}\left(x_{2}+k_{1}x_{1}\right). \end{array}$$

Consequently, control system (3) with control laws (21) and (24) can be written in the form

(25) $$\begin{array}{c} {\dot{\eta }}_{2}=A_{2}\eta_{2}, \end{array}$$

where

(26) $$\begin{array}{c} A_{2}=\left[\begin{array}{ccc} F & k_{1}\frac{g_{0}}{g}FG & -\frac{g_{0}}{g}FG\\ 0_{1\times M} & -k_{1} & 1\\ -\frac{g_{0}}{g}\psi & -k_{1}\left(\psi G+k_{1}\right) & \psi G+k_{1}+\frac{g_{0}}{g}k_{2} \end{array}\right], \end{array}$$

with 1 × *M* zero matrix 0_1×*M*_.

(*2) Case n* = 3. Similar as above, a vector of state variables is set as

(27) $$\begin{array}{c} \eta_{3}=\left[\begin{array}{c} e_{\xi }+\frac{g_{0}}{g}x_{3}G\\ x_{1}\\ x_{2}+k_{1}x_{1}\\ x_{3}+k_{1}x_{2}+k_{2}\left(x_{2}+k_{1}x_{1}\right) \end{array}\right], \end{array}$$

where *k*_1_, *k*_2_, *k*_3_ are tuning parameters of the feedback law

(28) $$\begin{array}{c} w=-k_{3}\left(x_{3}+k_{1}x_{2}+k_{2}\left(x_{2}+k_{1}x_{1}\right)\right). \end{array}$$

Control system (3) with control laws (21) and (28) yields a system that can be put in the form

(29) $$\begin{array}{c} {\dot{\eta }}_{3}=A_{3}\eta_{3}, \end{array}$$

where

(30) $$\begin{array}{c} A_{3}=\left[\begin{array}{cccc} a_{11} & a_{12} & a_{13} & a_{14}\\ a_{21} & a_{22} & a_{23} & a_{24}\\ a_{31} & a_{32} & a_{33} & a_{34}\\ a_{41} & a_{42} & a_{43} & a_{44} \end{array}\right], \end{array}$$

with

(31) a11=F  a12=−k12g0gFGa13=(k1+k2)g0gFG  a14=−g0gFGa21=01×M  a22=−k1  a23=1a24=0  a31=01×M  a32=0a33=−k2  a34=1  a41=−g0gψ  a42=k12(ψG+k1)a43=−(k1+k2)(ψG+k1)−k22a44=ψG+k1+k2+g0gk3.

Straightforward calculations show that matrices *A*_2_ (26) and *A*_3_ (30) are Hurwitz matrices for appropriate selections of *k*_1_, *k*_2_ and *k*_1_, *k*_2_, *k*_3_, respectively.

As a result, the state **x**(*t*) of control system (3) is guaranteed to asymptotically decay to a small neighbourhood of the origin if dynamic feedback controller (21) that consists of dynamic fuzzy logic controller (20) and feedback law (24) or (28) is implemented. Since the controller relies only on the nominal functions *f*_0_ and *g*_0_ and estimates the uncertain and unknown variables by processing the system state, it is a robust controller.

## 4. Simulation Results

In this section, a magnetic levitation system in which an electromagnet exerts attractive force to levitate a steel ball is considered. According to [27], the system dynamics can be represented by

(32) x˙1=x2x˙2=x3x˙3=f(x)+g(x)u+d(t),

where

(33) f(x)=f1(x)+f2(x),f1(x)=2(g−x3)(X∞+x1)x2,f2(x)=−2(g−x3)Qx2−R(X∞+x1)2(X∞+x1)(Q+L∞(X∞+x1)),g(x)=−2MQ(g−x3)M(Q+L∞(X∞+x1)),d(t)=g(x)δ(t).

In (33), we have the following:

*x*_1_: air gap (vertical position) of the steel ball, 0 m ≤ *x*_1_ ≤ 0.013 m,
*x*_3_: coil current,
*g*: gravity acceleration, 9.8 ms^−2^,
*M*: mass of the steel ball, 0.54 kg,
*R*: electrical resistance, 11.88 Ω,
*u*: voltage control input, −60 V ≤ *u* ≤ 60 V,
*L*_*∞*_: coil constant, 0.7987 H,
*Q*: magnetic core constant, 0.001624 Hm,
*X*_*∞*_: steel ball constant, 0.008114 m,
*δ*(*t*): bounded external disturbance, *δ*(*t*) = 5sin(*π*/2*t*).

**Figure 2:**
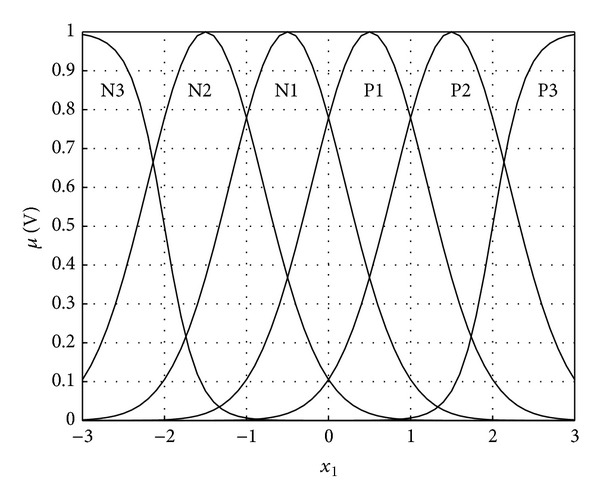
Membership functions of *x*_1_, *x*_2_, and *x*_3_.

In this Matlab simulation exercise, dynamic feedback controller (21) with feedback law (28) is applied to magnetic levitation system (32) to verify its performance as a robust stabilizer. The control objective is to drive the system state from **x**(0) = [0.0013 0 0]^*⊤*^ to the origin. The nominal functions *f*_0_(**x**) and *g*_0_(**x**) are obtained by replacing the system parameters in (33) with nominal values as listed in Table 1. The nominal system parameters with considerable errors are used in the controller to demonstrate its robust performance. The membership functions of *x*_1_, *x*_2_, and *x*_3_ are depicted in Figure 2.

**Figure 3:**
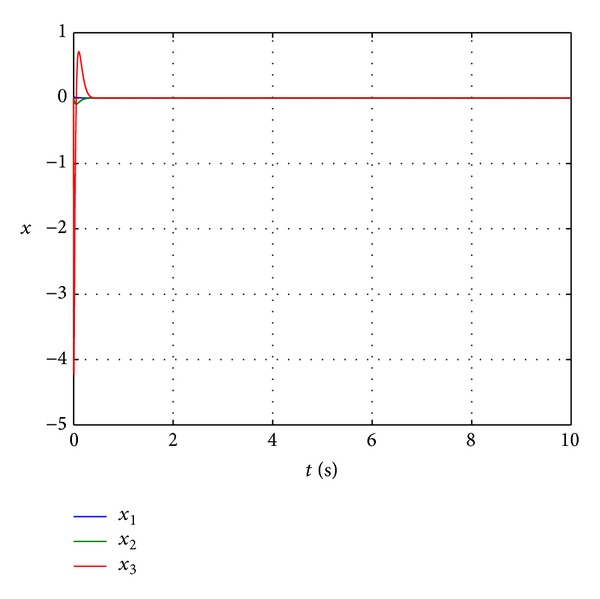
Trajectory of system state **x**.

**Figure 4:**
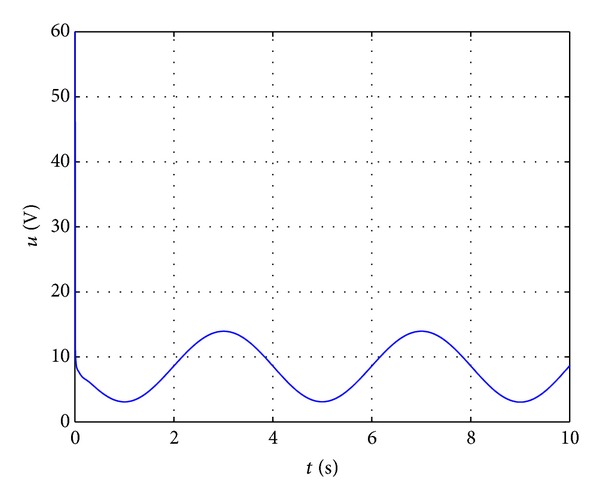
Control input *u*.

**Figure 5:**
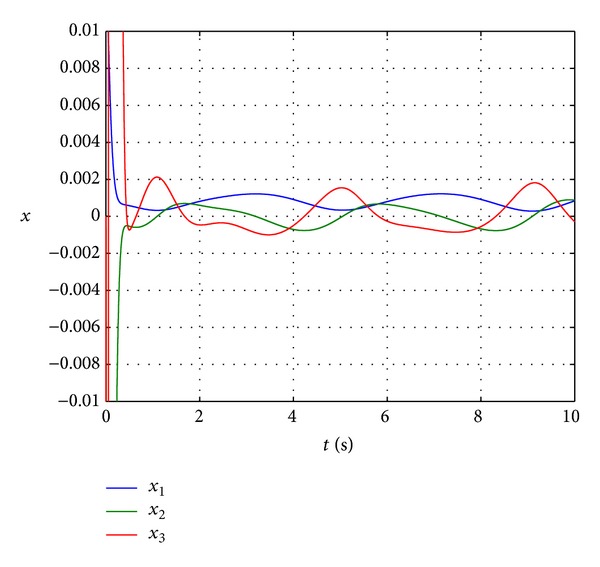
Trajectory of system state **x**.

Figures 3 and 4 show the state trajectory **x** and control input *u* of nonlinear system (32) for simulation time *t* = [0,10] s. In Figure 3, it can be seen that dynamic feedback controller (21) with feedback law (28) is effective in navigating the system state from **x**(0) = [0.0013 0 0]^*⊤*^ to the origin, even though only *f*_0_(**x**) and *g*_0_(**x**) are known and *d*(*t*) is not measured. To ensure that the control input is within −60 V ≤ *u* ≤ 60 V, a saturation block is added in the Simulink model.  Figure 4 illustrates that the control input *u* is well within the physical constraint. A closer examination of the state trajectory plot reveals that **x** does not settle exactly at zero but converges to a small neighbourhood of the origin as depicted in Figure 5. The reason for this behaviour is the presence of the persistent disturbance *d*(*t*) that affects the control system. Nevertheless, the state response of the magnetic levitation system controlled by *k*(**x**, *t*) with (28) fits the definition of robust asymptotic stability [28, 29].

## 5. Conclusion

A robust fuzzy logic stabilizer is proposed for nonlinear control systems with disturbances. The dynamic feedback controller design combines a control law to compensate for nonlinear terms in a system to be controlled and a dynamic fuzzy logic controller to handle unknown model uncertainties and disturbance. The controller construction assumes that only nominal functions of a control system are known, measurements of system state are available, and the disturbance is not measured. A tuning parameter of the fuzzy logic controller is adjusted by means of a linear time-varying system with state measurements as its input. The proposed controller is implemented in a magnetic levitation system and simulation results verify its capacity as a robust stabilizer. Equipped with its mathematically proven robustness property, the controller manages to regulate the system state asymptotically to a small neighbourhood of zero. To validate its control performance, the implementation of the robust fuzzy logic stabilizer in an experimental DC motor tracking system is considered as a future work.

## Figures and Tables

**Table 1:** Nominal parameters of the magnetic levitation system.

*g*_0_	9.0	ms^−2^
*M*_0_	0.30	kg
*R*_0_	10.0	Ω
*L*_∞0_	0.50	H
*Q*_0_	0.0003	Hm
*X*_∞0_	0.0020	m
